# A cross-immunization model for the extinction of old influenza strains

**DOI:** 10.1038/srep25907

**Published:** 2016-05-13

**Authors:** Florian Uekermann, Kim Sneppen

**Affiliations:** 1 Niels Bohr Institute, University of Copenhagen, Denmark

## Abstract

Given the frequent mutation of antigenic features, the constancy of genetic and antigenic diversity of influenza within a subtype is surprising. While the emergence of new strains and antigenic features is commonly attributed to selection by the human immune system, the mechanism that ensures the extinction of older strains remains controversial. To replicate this dynamics of replacement current models utilize mechanisms such as short-lived strain-transcending immunity, a direct competition for hosts, stochastic extinction or constrained antigenic evolution. Building on the idea of short-lived immunity we introduce a minimal model that exhibits the aforementioned dynamics of replacement. Our model relies only on competition due to an antigen specific immune-response in an unconstrained antigenic space. Furthermore the model explains the size of typical influenza epidemics as well as the tendency that new epidemics are associated with mutations of old antigens.

The main question we explore is: What are the minimal requirements for a dynamics where old influenza variants go extinct when new antigenically different strains of the same subtype emerge?

The antigenic features of the influenza virus evolve sufficiently fast to allow the virus to escape the acquired immune memory of an individual over the course of a few years. As a result, different strains that share some antigenic features emerge frequently. The interaction of antigens with the hosts immune network^1^, as well as the spreading dynamics of multiple strains which exhibit partial cross-immunization^2^,^3^,^4^,^5^,^6^ have been popular topics in recent research. Surprisingly, the observed antigenic change did not result in an exponential increase in the diversity of coexisting antigenically different strains. On the contrary, co-circulating strains of the same subtype are usually closely related^7^, and the dominating cluster of antigenically similar strains is replaced every 2–5 years^8^,^9^. The various aspects of this dynamics have been studied using models with varying degrees of detail and abstraction^10^,^11^,^12^,^13^,^14^,^15^,^16^,^17^.

Ferguson *et al*.^12^ introduced a detailed model which reproduced many patterns of influenza evolution, including the replacement of old strains by new ones, while at the same time avoiding extinction. Their model incorporates a long lasting cross-immunization between closely related strains, supplemented by a short-lived non-specific immunity to any influenza strain. A minimal version of this mechanism was considered by Tria *et al*.^13^. In both cases the hypothesized general immunity leads to competition between all strains, which in turn limits the diversity.

Koelle *et al*.^11^ use a genotype-phenotype map to account for the differing antigenic effect of genetic mutations. Their model reproduces the main features of influenza without assuming a strain-transcendent immunity. However it leads to extinction in the simulated population, which in turn is mitigated by an influx of strains that are derived from recently prevalent strains. Another interesting use of genotype-phenotype mapping based on epistatic effects was proposed by Tria *et al*.^10^. However, this model enforces an upper limit of one simultaneous infection per host. This forces all strains to compete for hosts and thereby prevents unlimited growth of overall diversity.

Bedford *et al*.^15^ constrain the antigenic feature space to 2 dimensions and use a distance dependent cross-immunization to reproduce observed dynamics of antigenic influenza evolution. The dynamics of disease interaction in constrained antigenic space have been explored in previous work^16^,^17^,^18^.

Many of the models mentioned above are able to reproduce a diverse set of features of influenza in remarkable detail. In the interest of simplicity we will focus mainly on the qualitative feature of limited diversity without complete extinction in the context of rapidly changing antigenic features. In contrast to previous models we constrain neither the accessible antigenic feature space, nor the capacity of the system to sustain long-term survival of arbitrarily many strains. Our minimal influenza model, contains no competition between strains, except for specific (cross-)immunization, mediated by mutating antigens. The model exhibits the described substitution dynamics without leading to complete extinction.

Building on the idea of an immune response with short- and long-lived components we employ a long-lasting immune memory, and a short-lived immunity due to high antibody prevalence in the host. Both of these immune response components in our model specifically target an antigen exposed by the virus. We minimize our assumptions about the evolutionary constraints of the antigenic phenotype by using a discrete set of antigens per virus. Cross-immunization between strains is only possible if exactly the same antigen is present in both viruses and a host is immunized to that particular antigen.

Since only antigenic features of the virus influence the dynamics in our model, we are investigating the evolution of antigenic clusters instead of individual strains. From here on we use the term “strain” to refer to a class of antigenically equivalent viruses, which may differ genetically.

We aim to provide a qualitative understanding of the effects of multiple independent antigens in the presence of cross-immunization for influenza A/H3N2 and similar viruses.

## Model

We use a continuous-time agent-based model with a host population of well-mixed individuals. For simplicity we model all events and durations using rates, which we usually specify by their average waiting time.

Each individual can be infected by one or more strains at the same time. All strains are described by a set of 5 antigens^19^,^20^,^21^. In our model the immune response of a host always targets a specific antigen. Thus, the competition between strains is mediated by the immune response to their antigens. As a result, strains can only interact and compete if they share one or more antigens. The following paragraphs describe the individual hosts immune response, spreading of viruses between hosts and antigenic mutation in our model.

## Immune Response

**Figure 1:**
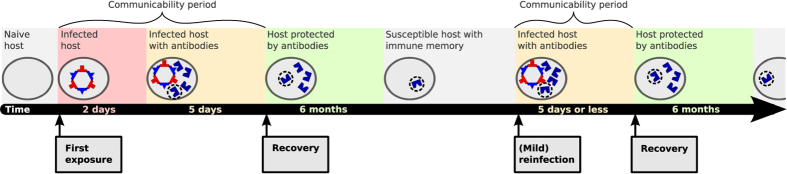
Stages of an infection and later reinfections. If a host is infected with an unknown strain (no immune memory the strain’s antigens, red background), it gains a high level of antibodies against one antigen of the strain after on average 2 days (yellow). The host recovers on average 5 days later (green). The host maintains high levels of the antibody for on average half a year, which protects it against reinfection with any strain with the corresponding antigen (green). After loosing this protective effect (gray), the host will keep immune memory to the antibody, but memory does not protect against reinfection. Upon reinfection, with a strain whose antigens match any immune memory of the host, the host will immediately produce high levels of this antibody (yellow). Thus, the host skips the infected period without antibodies when reinfected by strains with a familiar antigen. The subsequent infected period with antigens (yellow) and the protected period (green), are modeled identically for infections with unknown strains and reinfections. Notes: All stated periods lengths are averages, which are modeled using transition rates. The recovery rate is proportional to the number of different kinds of antibodies present in the host which match an antigen. However, a host will never develop new antibodies from an infection, if it already has one that matches an antigen of the strain (original antigenic sin, see Fig. 2b).

An average of 2 days after an infection a host will produce high levels of antibodies against one antigen of the infecting strain^22^. On average the host will recover from the infection 5 days later^22^. If multiple kinds of matching antibodies are present in the host, the recovery rate increases proportionally to their number. The presence of antibodies during and after the infection protects the host from being infected by any strain, which exhibits the antigen targeted by the antibody. This protective effect is lost after on average half a year, after that the antibody concentration is assumed to be too low to prevent reinfection completely^23^,^24^,^25^. However, the host will retain memory of the antigen (or antibody). Upon (re)infection with any strain that exhibits this antigen, it will immediately produce high levels of matching antibodies (skipping the initial phase of an infection with a completely unknown strain). Figure 1 contains a visual representation of this cycle of (re)infection, protection and susceptibility.

**Figure 2:**
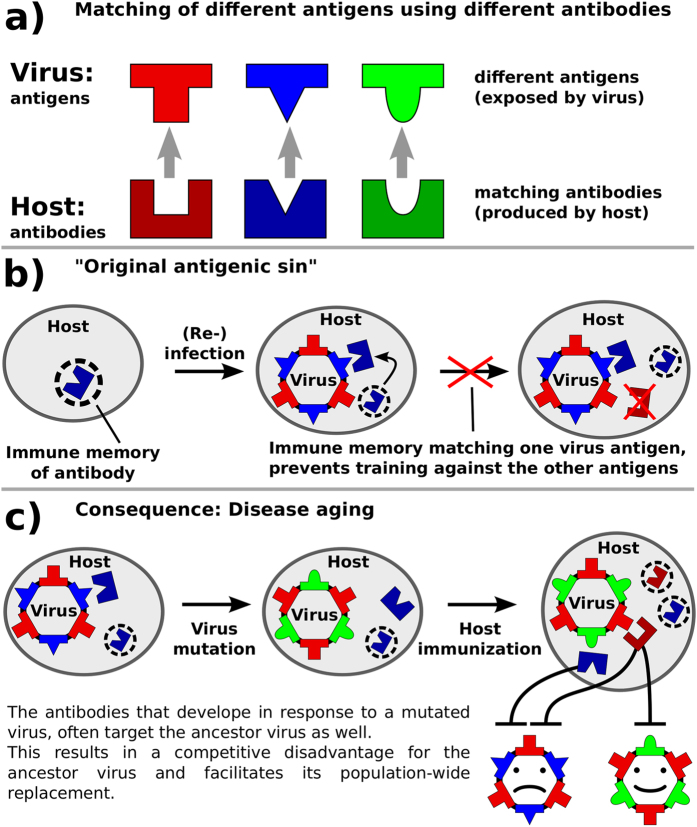
Key concepts of the model. (**a**) The same antigens are exposed by all viruses of the same antigenic cluster. The host immune system produces antibodies, which match a particular antigen, allowing the host to recover from infections and protect itself against reinfection with strains that expose the same antigen. In our model host-strain interaction and strain competition is exclusively caused by antigen-antibody interaction. (**b)** In our model, a host which has produced antibodies in response to a previous infection, will produce the same antibodies again, if it encounters any strain that exposes the matching antigen (immune memory). Furthermore, a hosts will not produce different antibodies to an infection if it has immune memory of other matching antibodies from previous infections (original antigenic sin^26^,^27^). (**c)** As a consequence of the concept of original antigenic sin, a host immune system usually targets only one antigen of a strain. However, infection with mutants allow the host to additionally target other antigens (shared by ancestor and mutant). This leads to a competitive disadvantage for old strains compared to new antigenic mutants.

Note: If a host has immune memory matching an antigen of an infecting strain, it will not acquire antibodies matching a different antigen from this infection (see Fig. 2a,b). This resembles the original antigenic sin^26^,^27^ and has the consequence that immune systems in the population only “learn” when a new strain is introduced.

## Spreading and Mutation

The spreading rate is chosen such that the basic reproduction number is *R*_0_ = 3. That means a host without preexisting immune memory will expose on average 3 other hosts to the virus before recovering. If the exposed host does not have antibodies against the transmitted virus, it will be infected, which triggers the immune response described above. The spreading rate of first infections and reinfections is the same. Therefore, reinfections expose fewer hosts to the virus on average.

In our model a strain will antigenically mutate inside a host with a rate of 0.5/*N* per day. There are *N* = 512000 hosts in the system. During mutation one of the 5 antigens of the ancestor strain is replaced by a new antigen. The new antigen is completely unrelated to any other antigens in the system.

If the host has no antibodies against the mutated strain, it will be infected and the virus will potentially be transmitted to other hosts in the system (like any other infection). If the host does have any antibodies against the mutated strain, the strain is eliminated immediately. Due to the highly abstracted mutation mechanism we base our choice of mutation rate on the desired time between cluster transitions.

## Results

**Figure 3:**
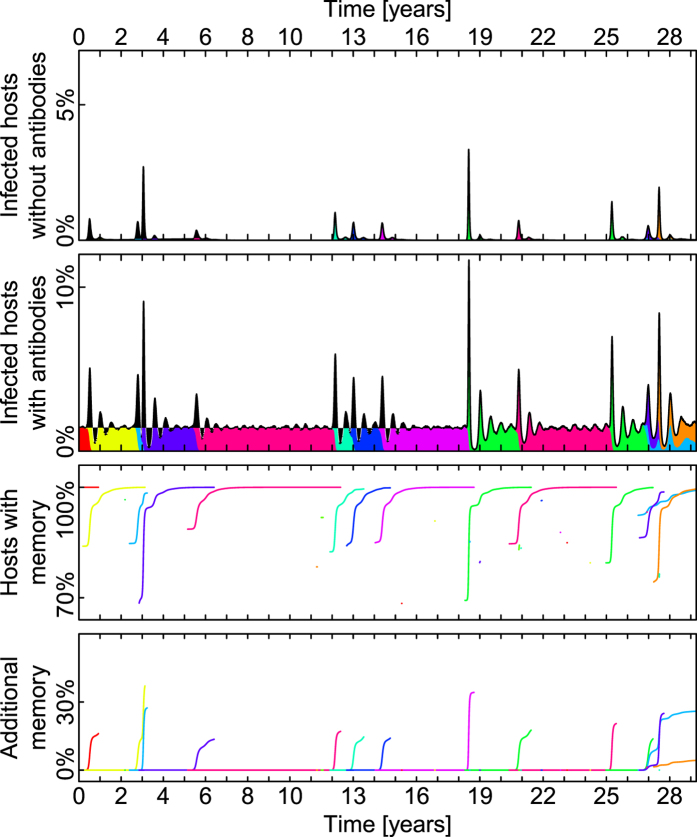
Typical behavior of the model (*R*_0_ = 3, *N* = 512000 hosts, seeFig. 1 for other parameters). Each color represents a different antigenic cluster. Panels (top to bottom): (1) Percentage of the population infected by a strain unknown to their immune system (no matching immune memory). Corresponds to red stage in Fig. 1. (2) Percentage of the population which is infected and has antibodies matching at least one of the strain’s antigens. Corresponds to yellow stage in Fig. 1. (3) Percentage of hosts, that have immune memory to the antigenic cluster. (4) Additional immune memory, on top of immune memory shown in panel (3).

Our main objective is to model the cluster substitution, without resorting to introducing antigen independent immune system mechanisms. Figure 3 shows a typical evolution of the system over the span of three decades. The following paragraphs focus on the cycle of cluster substitution.

### Behavior between cluster substitution

An important dynamical feature of our model is that the protection due to high antibody concentration is lost some time after recovery. Since immune memory, which is never lost, does not protect against infections, hosts eventually become susceptible to any strain again. This means that diseases can survive in a population with immune memory against the disease, a feature that prevents the extinction of the investigated class of diseases.

If a host is exposed to a known strain, it will immediately produce antibodies matching the antigens it has immune memory to, thus skipping the immunization process, which leads to faster recovery, but prevents immunization to other antigens (original antigenic sin^26^,^27^).

If no mutations happen and only one strain is present in the system, the dynamics quickly approaches that of a traditional SIRS system, which have a globally stable endemic equilibrium state for a broad range of parameters.

We assume most of these reinfections to be asymptomatic or mild^24^,^25^,^28^. In our model they serve mainly as a reservoir to prevent complete extinction of influenza and as a source of mutations. These reinfections are shown in Fig. 3b (each color represents a strain). During periods where only one strain (one color) is present, the fraction of the system being infected is nearly constant.

### Antigenic mutation and infection wave

The process of one antigenic cluster (here referred to as strain) being replaced by a successor starts with an antigenic mutation. The new strain escapes the immune memory of some hosts. The hosts whose immune memory the new strain escapes are those which acquired immune memory to the one antigen which was present in the ancestor strain, but is not present in the new mutated strain. This allows the new strain to spread rapidly in the subpopulation that has no immune memory to any of it’s antigens. This rapid spread is visible in Fig. 3a as spikes of infections. We assume that these first infections of defenseless hosts constitute most of the 1 week long symptomatic infections that are typically associated with influenza^22^.

Figure 3c shows how many hosts have immune memory to at least one antigen of a disease (line colors correspond to cluster). For the ancestor cluster at the time of an antigenic mutation this is usually close to 100%, because most hosts have immune memory to one of its antigens. At the time of mutation this number is lower than 100%. This difference represents the percentage of hosts whose immune memory the new cluster escaped by mutating an antigen. The immune memory acquired in the time following the emergence of a new cluster is visible here as the quick increase of the line in the color representing the new cluster.

The fraction of individuals experiencing these first time infections fluctuates around 20% (tendency to greater values, see next paragraph). The exact number varies depending on the history of the antigen that was replaced in the mutation event. This number is consistent with the measured number of symptomatic infections observed in a typical pandemic following the emergence of a new antigenic cluster and a observed typical cross-immunity of 80% or less between subsequent dominant clusters^29^,^30^. The typical size of the wave of first infections after a cluster replacement is strongly coupled to the number of antibodies in our model (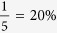).

Due to their longer presence in the system, old antigens, that have been present in the dominant antigenic clusters for longer time, are targeted by more host immune systems (see next paragraph for details). Since escaping the protective effect of present antibodies is necessary for a mutation to spread, those mutations that mutate these old antigens are less likely to go extinct during their first days in the system. This favors the survival of mutations in different epitopes than the ones that mutated recently, a pattern observed in influenza A^9^.

### Immunization against new cluster and replacement of old cluster

Upon first infection with strains of the new cluster the infected hosts acquire new antibodies and immune memory to exactly one antigen. With probability 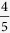 the targeted antigen will be one that is present in strains of both the new and the old antigenic cluster (see Fig. 2c). In this case the host has acquired a second kind of immune memory to the ancestor strain. Figure 3d shows this additional immune memory. Here only hosts are counted that have immune memory to two or more antigens of the cluster represented by the corresponding color. Note that an increase in additional immune memory is only visible for the old cluster.

In hosts that acquired additional immune memory to the old strain, this results in a lower spreading efficiency of strains of the old cluster for 2 reasons: (1) The additional immune memory shortens the average time until recovery (the time until recovery is calculated independently per antibody, the shortest one matters). This shortens the time during which it can be transmitted to other hosts. (2) A recovered host with protective antibodies can not be infected by strains of the old cluster until both antibody levels declined sufficiently.

Due to sharing 4 antigens, the strains of the new mutated cluster and those of the old ancestor cluster are in direct competition in most of the host population: A host infected by one of them will often be removed from the susceptible compartment for both of them. Coupled with the less efficient spreading of strains of the old cluster, this competition leads to it’s elimination. This is visible in Fig. 3b as a decline in infections with the old strain and it’s eventual extinction.

In short, as summarized in Fig. 6, the ancestor cluster seals its fate by mutating an antigen and generating offspring that has both an initial competitive advantage (first infection wave) and a long term one (additional immune memory against old cluster).

### Growth of diversity

Two disease strains that share no antigens, would spread completely independent from each other. There is no direct density dependent competition to limit the diversity of strains or total number of infections. This is a key difference between our model and those which incorporate non-specific immune-response components^12^,^13^.

This complete lack of interaction between antigenically independent strains inevitably leads to exponential growth of diversity of strains in the long run. But, for the timescale on which the cycle of antigenic drift and cluster substitution is observed within a subtype, the dynamics shown in Fig. 3 exhibits the desired replacement dynamics.

**Figure 4:**
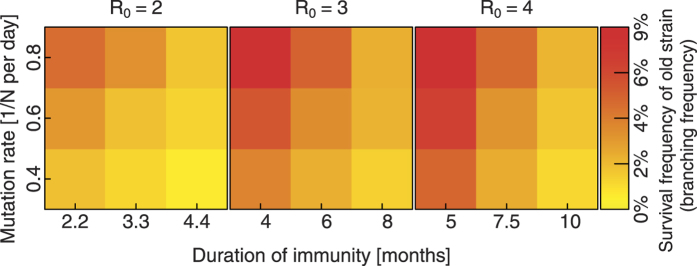
Survival frequency of old strain if a new strain emerges. This is equivalent to the splitting frequency of the lineage per generation. Each data point is the result of 10000 sucessful mutations (one or more infections) in a system with 500000 hosts. The duration of immunity (antibodies) is chosen such that the average number of infected hosts (and thus generation time) in the center of each panel are similar. The parameters in the center of the middle panel (*R*_0_ = 3) are the same as in Fig. 3.

Figure 4 shows how often the lineage splits into two separate branches instead of the new strain replacing the old one, for a variety of different parameters. For the parameters used in Fig. 3 the measured frequency of branching is (3.8 ± 0.3)%. In a system without cross-immunization (one antigen) the branching rate would be 100%.

The branching frequency increases with mutation rate and *R*_0_, and decreases with a longer duration of immunity. The number of mutations in the system and thus the generation time, depend on the number of infected hosts between cluster transitions and the mutation rate. Figure 4 indicates that decreasing mutation rate and increasing duration of immunity would decrease branching rates even further. This would however require more computational resources, because the resulting decrease in the fraction of infected hosts, necessitates a larger host population, to prevent extinction of strains due to fluctuations.

### Prediction of antigen substitution

In our model, a successful cluster substitution requires that the mutated strain evades the immune system of it’s first host and spreads successfully before recovery. The probability of evasion and successful spreading depend on the immune status of the population regarding that antigen. Thus, some antigens of the current dominant antigenic cluster are more likely to be replaced during the next cluster substitution. The likelihood of a certain antigen mutation resulting in such a substitution is determined by the evolutionary history of the virus, which shaped the immune status of the population. This allows a prediction of which antigen will mutate during the next cluster substitution from an evolutionary history of the system.

**Figure 5:**
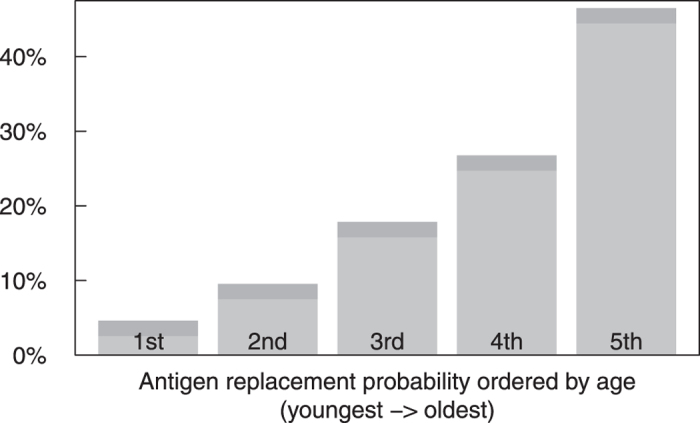
Probability of replacement of each antigen during the next cluster substitution ordered by age. 1st represents the antigen mutated during the previous cluster substitution, 2nd the next oldest one, etc. Probabilities are inferred from the simulation shown in Fig. 3 after 10000 successful mutations (one or more infections). Strains which die out in less than 50 days are not counted. The dark grey area represents the 1*σ* confidence interval.

**Figure 6:**
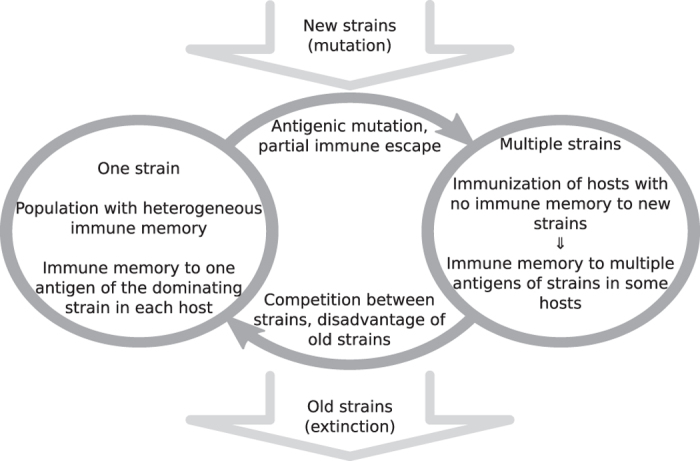
Schematic of the cycle alternating between one strain steady state and transitional periods of mutation and replacement.

By using the order in which the antigens of the currently dominating cluster evolved, we can estimate which antigen will be replaced next (see Fig. 5). In particular, the antigen that mutated during the last cluster substitution will not be the next one to be replaced next with a probability of >90%. Such behavior has in fact been observed in influenza A evolution, where subsequent cluster transitions are typically dominated by changes at different antigenic sites^9^.

## Discussion

In this paper we introduced a minimal model for the infection dynamics of influenza in the presence of antigenic drift. Utilizing the well established concepts of cross-immunization and original antigenic sin, our model reproduces the dynamics of antigenic cluster substitution. Building on the idea of strong short time cross-immunity as primary source of competition between strains^12^,^13^, we differentiate between short time immunity and long lasting immune memory. However, in contrast to the established models utilizing short term immune response effects^12^,^13^, both short and long term effects target specific antigens in our model and have known biological counterparts (memory B cells and antigens). This allows us to use known features of the adaptive immune system as motivation for all crucial ingredients of our model, that lead to the desired substitution dynamics of antigenic clusters. While Bedford *et al*.^15^ achieve the same effect with a disease specific one component immune-response, their model requires small antigenic mutations between cluster transitions to sustain the virus population. This leads to a more complex model of cross-immunity declining with distance in an antigenic space limited to 2 dimensions, in which differently sized steps reflect antigenic phenotype mutations.

An unconventional feature of our model is the possibility for reinfection of hosts with immune memory matching an antigen of the infecting strain^23^,^24^,^25^. This assumption generally favors the survival of strains and matches the observation of long lasting survival of aging antigenic clusters until a new cluster appears by mutation. In a more realistic model the reinfection frequency could be reduced by considering fine grained mutations within clusters, birth of new hosts, or a structured population.

Since we aim at providing a minimal mechanism for cluster substitution in influenza, our model does not reproduce all observed features of influenza epidemics accurately. In particular, the lack of geographic structure weakens the effect of herd immunity, which would lower the fraction of infections when a new cluster emerges. This and immune memory from earlier infections will lead to an observed reproduction number that is lower than *R*_0_. Another simplification we introduce is the punctuated replacement of antigens with a new one, which is not targeted by antibodies matching the old antigen. This mutation mechanism allows us to simulate the spreading of antigenic clusters instead of individual genetically identified strains. A more realistic approach would be a gradual decrease in cross-immunity based on a genotype to antigenic phenotype mapping^10^,^11^,^15^,^16^,^17^,^18^. Such an approach could enhance the fitness disadvantage of old strains in general and support the prevention branching. However we found that our simple model of antigenic mutation proved to be sufficient for reproducing the desired dynamics and requires fewer assumptions on how exactly the antigenic feature space is traversed and the mapping of antigenic distances to cross-immunity. Future extensions of our model, implementing such a gradually diminishing antibody-antigen affinity would allow a relaxation of the concept of original antigenic sin to single antigenic sites, at the expense of higher model complexity.

The key feature of our model is the antigen specific immune system, which couples the long-lived immune memory with the short-term immunity and disease replacement. Our model directly implies different history dependent probabilities for each antigenic site to successfully mutate and start an antigenic cluster substitution. A history dependence which, as shown in Fig. 5, implies a strongly enhanced effective mutation rate for the oldest antigens on the surface of the virus. We want to stress that the replacement dynamics of influenza spreading and evolution can be replicated with only a specific immune response combined with a set of antigenic features, that each can be targeted by the immune system and mutate independently.

## Additional Information

**How to cite this article**: Uekermann, F. and Sneppen, K. A cross-immunization model for the extinction of old influenza strains. *Sci. Rep.* **6**, 25907; doi: 10.1038/srep25907 (2016).
